# Anticonjugation and Antibiofilm Evaluation of Probiotic Strains *Lactobacillus plantarum* 22F, 25F, and *Pediococcus acidilactici* 72N Against *Escherichia coli* Harboring *mcr*-1 Gene

**DOI:** 10.3389/fvets.2021.614439

**Published:** 2021-06-11

**Authors:** Prasert Apiwatsiri, Pawiya Pupa, Jitrapa Yindee, Waree Niyomtham, Wandee Sirichokchatchawan, Kittitat Lugsomya, Asad Ali Shah, Nuvee Prapasarakul

**Affiliations:** ^1^Department of Veterinary Microbiology, Faculty of Veterinary Science, Chulalongkorn University, Bangkok, Thailand; ^2^College of Public Health Sciences, Chulalongkorn University, Bangkok, Thailand; ^3^Jockey Club College of Veterinary Medicine and Life Sciences, City University of Hong Kong, Kowloon Tong, Hong Kong; ^4^Diagnosis and Monitoring of Animal Pathogens Research Unit (DMAP), Bangkok, Thailand

**Keywords:** probiotic, *Escherichia coli*, colistin resistance, *mcr* gene, anti-conjugation, anti-biofilm

## Abstract

Several species of lactic acid bacteria (LAB) are commonly used as probiotics and as an alternative to antibiotics in various industries, especially in the livestock industry. This study aimed to investigate the anticonjugation and antibiofilm activity of cell-free supernatant (CFS) of Thai LAB strains (*Lactobacillus plantarum* 22F, 25F, and *Pediococcus acidilactici* 72N) against colistin-resistant *Escherichia coli* isolates. A total of six colistin-resistant *E. coli* strains were isolated from different sources, including pigs, farmers, and farmhouse environments. The *E. coli* were characterized by plasmid profiling, PCR detection of *mcr*-1 gene, and antibiotic susceptibility patterns. The CFS at dilutions ≥1:16 was chosen as the proper dilution for anticonjugation assay. Besides, it could significantly reduce the transfer frequencies of resistance gene *mcr*-1 up to 100 times compared to the neutralizing CFS (pH 6.5). The biofilm production in the planktonic stage was reduced by non-neutralizing and neutralizing CFS determining with crystal violet staining assay up to 82 and 60%, respectively. Moreover, the non-neutralizing CFS also inhibited the biofilm formation in the sessile stage up to 52%. The biofilm illustration was confirmed by scanning electron microscopy (SEM). These results agreed with the findings of the crystal violet technique, which showed a significant reduction in cell density, aggregation, and extracellular polysaccharide (EPS) matrix. The application of Thai LAB may serve as an attractive alternative to antibiotics for reducing biofilm formation and limiting the proliferation of antibiotic-resistant genes.

## Introduction

Antimicrobial resistance (AMR) is one of the serious global health concerns that threaten both animal and human survival. The increase in resistance has made it challenging to treat such type of infections caused by antibiotic-resistant bacteria. Such bacterial infections can lead to ineffective treatments, higher treatment costs, and mortality in humans and animals. By 2050, antimicrobial-resistant pathogens could cause 10 million deaths annually with an expected cost of $100 trillion (1). Antibiotics are widely used as a feed additive in livestock production to improve growth performance and combat several infections. Colistin is used as a last resort for the treatment of multiresistant bacterial infections not only in humans but also in animals, especially in swine (2–4). The emergence of plasmid-mediated colistin resistance encoded by the *mcr*-1 gene in *Escherichia coli* isolates of pigs, chickens, and humans has raised global concern about the potential horizontal transfer of this gene between humans and animals (3). Therefore, the rapid spread of colistin-resistant *E. coli* has been found in many countries, and more than 10 additional gene homologs of *mcr* have been identified since then (3, 5). Worldwide, there are more reports of *mcr*-mediated resistance in animals when compared to human isolates, suggesting that plasmid-mediated colistin resistance is more prevalent in livestock (6).

Bacterial biofilms, the polymeric substances secreted by microbes, are one of the main resistance mechanisms that bacteria use to survive against various stresses, including antibiotics, disinfectants, and host defenses (7). Biofilms decrease the activity of antimicrobial agents by trapping and preventing them to reach the target sites. Besides, most of the biofilm-forming bacteria are less active metabolically, which could reduce the efficacy of antibiotics, which are effective against active dividing cells (8). Consequently, microbial biofilms present a severe medical problem and contribute to the development of chronic and recurrent infections in both humans and animals. Therefore, there is an urgent need to find alternative therapies that can overcome these challenges.

Recently, food-based probiotics have assumed great significance for their nutritional and therapeutic potential (9). Probiotics are defined by the World Health Organization (WHO) as “live microorganisms which, when administered in adequate amounts, confer a health benefit on the host” (10). Probiotics have been categorized by genus, species, and strain, for example, *Lactobacillus rhamnosus* GG. Studies have shown that physiological benefits of probiotics are strain-specific since different strains of the same species can have different health effects (9, 11). During the past few decades, lactic acid bacteria (LAB), a popular member of probiotics, have been extensively used in humans and animals for various purposes to enhance nutrient utilization, to modulate both the innate and the adaptive immune systems, and to inhibit the growth of numerous pathogenic microorganisms (12, 13). Besides, LAB strains have been shown to limit the emergence of bacterial resistance by inhibiting the horizontal transmission of resistance genes (14, 15) and biofilm production (16, 17). The LAB produces several active metabolites, including organic acids, bacteriocins, hydrogen peroxides, exopolysaccharides, and biosurfactants, all of which may prevent the formation of biofilms (9). Generally, most of the metabolites are secreted into a broth medium during the propagation of bacteria and known as a supernatant. The LAB supernatant exhibits anticonjugation and antibiofilm activity against various pathogens, as mentioned above. Based on our previous studies, *L. plantarum* 22F, 25F, and *Pediococcus acidilactici* 72N showed promising performance and strong antibacterial activity against enteric pathogens (18–20). However, their antibiofilm and anticonjugation potentials were not determined yet. To the best of our knowledge, this is the first report on the antibiofilm and anticonjugation activity of LAB using cell-free supernatant (CFS) against colistin-resistant *E. coli*.

In line with that, the objective of this study was to evaluate the anticonjugation and antibiofilm activities of CFS of different LAB species (*L. plantarum* 22F, 25F, and *P. acidilactici* 72N) against *E. coli* harboring *mcr*-1 gene from human, pig, and environmental origins.

## Materials and Methods

### Bacterial Strains

In our previous studies, *Lactobacillus plantarum* 22F, 25F, and *Pediococcus acidilactici* 72N were isolated in Thailand from antibiotic-free healthy commercial fattening and indigenous pigs (18). The LAB isolates displayed attractive probiotic properties, and their *in vitro* features make them potential candidates for probiotic applications (19, 20). In this study, six *mcr*-1 positive colistin-resistant isolates of *E. coli* were employed based on antimicrobial sensitivities, plasmid replication, biofilm formation, and the source (Table 1). These isolates were collected from feces or wastewater at a swine farm in the central part of Thailand. The biohazard execution control was approved by the Institutional Biosafety Committee of the Faculty of Veterinary Science, Chulalongkorn University (IBC 1731021). The samples were collected directly into a sterile container and transferred to a laboratory at 4°C. All samples were 10-fold diluted in sterile normal saline, and the dilutions at 10^7^-10^8^ were spread on Eosin Methylene blue agar (Oxoid, Hampshire, England, UK) supplemented with colistin (2 μg/ml: Sigma, St. Louis, MO, USA) for selecting colistin-resistant *E. coli*. A representative pure colony was selected randomly to detect the *mcr*-1 gene by using a specific primer as described previously (3). In addition, wild-type *E. coli* J53 was used as the recipient strain to examine bacterial conjugation (21). This strain is negative for fertility factors and resistant to sodium azide (MIC >512 μg/ml), and sensitive to colistin (MIC <2 μg/ml). All isolates used in this study were affirmed by the matrix-assisted laser desorption/ionization time-of-flight (MALDI-TOF) Biotyper (Bruker Daltonics, Billerica, MA, USA) according to the manufacturer's recommendation with a high-confidence identification score.

**Table 1:** Profiles of lactic acid bacteria and *Escherichia coli* strains used in this study.

**Bacteria**	**Isolate**	**Accession number**	**Antibiogram**	**Plasmid replicon**	**Colistin MIC (μg/ml)**	***mcr*-1 gene**	**Biofilm formation**	**Origin**	**Reference**
*Lactobacillus plantarum*	22F	LC035101	CST	ND	ND	ND	ND	Pig	(18)
	25F	LC035105	CST	ND	ND	ND	ND	Pig	
*Pediococcus acidilactici*	72N	LC035107	CST	ND	ND	ND	ND	Pig	
*Escherichia coli*	P01	NA	AMP-AMX- CEF- CHL-CLX-CPD- CST-ENRO-INN-MFX-NIT-PIP-SXT-TET	FIB, Frep, W	8 (R)	+	Strong	Pig	This study
	P02	NA	AMP-AMX-CLX- CHL-CST-PIP-SXT	Frep	16 (R)	+	Strong	Pig	
	H01	NA	AMP-AMX- CLX- CST-PIP-TET	FIB	4 (R)	+	Strong	Human	
	H02	NA	AMP-AMX-CEF-CHL- CLX-CPD-CST-INN- GEN-MFX-PIP-SXT-TET	FIB, Frep	8 (R)	+	Strong	Human	
	E01	NA	AMP-AMX-CHL-CLX-CST-ENRO-GEN-MFX- PIP-SXT-TET	FIB, Frep, Y	4 (R)	+	Strong	Environment	
	E02	NA	AMC-AMP-AMX- CEF-CHL-CLX-CPD -CST- ENRO-INN-GEN-MFX-PIP	FIB, Frep	4 (R)	+	Moderate	Environment	
	J53	NA	AMP-AMX	Not detected	<2 (S)	–	ND	NA	

*R represents resistance to colistin (MIC values are more than 2) and S represents susceptibility to colistin. Isolates of E. coli that showed positively to mcr-1 gene by using PCR are expressed as + (presence) or – (no presence). AMC, amoxicillin–clavulanic acid; AMP, ampicillin; AMX, amoxicillin; CEF, ceftiofur; CHL, chloramphenicol; CLX, cefalexin; CPD, cefpodoxime; CST, colistin; ENRO, enrofloxacin; ERY, erythromycin; KAN, Kanamycin; INN, Cefovecin; IPM, Imipenem; GEN, gentamicin; MFX, marbofloxacin; NA, no available; ND, no determined; NIT, nitrofurantoin; PIP, piperacillin; STR, Streptomycin; SXT, trimethoprim/sulfamethoxazole; TET, tetracycline; TYL, Tylosin*.

### Plasmid Replicon Typing

The genomic DNA of *E. coli* strain was extracted from an overnight culture using the GeneJET Genomic DNA Purification Kit (catalog no. K0721; ThermoFisher Scientific, Waltham, MA, USA) according to the manufacturer's recommendations. Plasmids were typed by the PCR-based replicon typing (PBRT) using the genomic DNA of the donors and transconjugants as template and primers described previously (22). PCR amplification was carried out with 18 pairs of primers recognizing FIA, FIB, FIC, HI1, HI2, I1-Iγ, L/M, N, P, W, T, A/C, K, B/O, X, Y, F, and FIIA in five multiplexes and three simplex reactions. PCR positive replicons identified in our previous studies were used as positive controls (23, 24).

### Preparation of *Lactobacillus* Cell-Free Supernatants

Cell-free supernatants (CFSs) were prepared as described previously with minor modifications (19, 20). Briefly, each LAB isolate at 10^8^ CFU/mL concentration was inoculated into 30 ml of MRS (de Mann Rogosa Sharpe) broth (Becton, Dickinson, and Company, Sparks Glencoe, MD, USA) left incubated at 37°C for 24 h. Subsequently, all CFSs were obtained by centrifugation for 10 min at 4,500 rpm and 4°C. The collected supernatants were separated into two groups, a cell-free fraction and a neutralizing fraction, where the latter was obtained by adjusting pH to 6.5 ± 0.1 using 1 M NaOH (Carlo Erba Reagents, Val de Reuil, France). Both fractions were filter-sterilized by 0.22-μm surfactant-free cellulose acetate filters (Corning, Corning, NY, USA).

### Preparation of CFS Dilution

In our previous study, three LAB strains had shown strong antibacterial activity against enteric pathogens (20). Therefore, the minimal bactericidal concentrations of CFSs of LAB against *E. coli* strains were evaluated before performing an antiplasmid conjugation assay. The *E. coli* strains were grown overnight at 37°C on Luria–Bertani (LB) agar that contains yeast extract 5 g/L, Tryptone 10 g/L (Becton, Dickinson, and Company, Sparks Glencoe, MD, USA), and NaCl 10 g/L (Carlo Erba Reagents, Val de Reuil, France). The pH of LB media was adjusted to 7.5. The harvested colonies were resuspended in LB broth and adjusted to 1.5 × 10^8^ CFU/ml. CFSs of *L. plantarum* 22F, 25F, and JCM1149 as the reference strain were serially diluted 2-fold (non-diluted, 1:2, 1:4, 1:8, 1:16, 1:32, and 1:64), where the diluted CFS at 1:64 reflected the same pH value with the neutralizing CFS. A 600 μl of CFS was added to equal amount of bacterial inoculum and incubated overnight at 37°C. The viable cells were then analyzed by measuring colony forming units (CFUs/ml) on the LB agar plates. The highest dilution without bactericidal effect was used to determine the plasmid conjugation rate. The experiments were performed in triplicates.

### Antiplasmid Conjugation and *mcr*-1 Gene Confirmation

To investigate the mechanism of action of LAB on gene transfer, experiments were performed on donor and recipient strains. The donor and recipient strains were cultured in LB broth and incubated at 37°C overnight. At an equal quantity, the donor and recipient strains were mixed in a sterile tube with the final concentration at log 7.5 CFU/ml. The bacterial suspension was added with CFS (1:16 dilution), neutralizing CFS, or CFS of *E. coli* ATCC 25922 as an internal control, while sterile LB broth was used as a negative control. Each assay was performed in triplicate. After incubation of 24 h, the suspensions were serially diluted 10-fold in sterile normal saline. Transconjugants were selected on LB agar plates supplemented with NaN_3_ (200 μg/ml: Oxoid, Hampshire, England, UK) and colistin (2 μg/ml) (Sigma, St Louis, MO, USA). This condition was also used in our preliminary study for examining the growth of both colistin-resistant and recipient *E. coli*. The results demonstrated that they could not grow on this selected medium. Therefore, only recipient *E. coli* (J53) receiving colistin-resistant gene from the donor *E. coli* could grow on this medium (data not shown). Transfer frequencies were determined by dividing the number of transconjugants by the number of donor colonies (log of transconjugants on selective media/log of the donor). The presence of *mcr*-1 in transconjugants was also screened using PCR, broth microdilution assay, and plasmid replicon typing. For PCR assay, at least three colonies of transconjugants were selected randomly and individually detected the *mcr*-1 gene as described previously (3). The colistin-resistant phenotypes of the transconjugants were determined by the broth dilution method, while *E. coli* ATCC 25922 was used as a control strain (25). The plasmid replicon types were also confirmed in the transconjugants using PBRT.

### Effects of CFS on Biofilm Formation

Biofilm-forming abilities were determined in microtiter plates using a crystal violet binding assay with minor amendment (26). In brief, 200 μl of 10^6^ CFU/ml of the overnight culture *E. coli* was thoroughly mixed with 100 μl of CFS and 100 μl of NCFS of LAB in a sterile microtiter plate (Corning, Corning, NY, USA) and incubated at 37°C for 24 h, whereas sterile MRS broth was used as a control. The non-adherent cells were then gently removed by washing twice with sterile distilled water (DW) and fixed with 200 μl of methanol (RCI Labscan, Bangkok, Thailand) for 15 min. The fixed cells were stained with 200 μl of 0.1% crystal violet (Carlo Erba Reagents, Val de Reuil, France) in distilled water for 5 min. Following treatment with 160 μl of absolute ethanol (Merck, Darmstadt, Germany), the stained cells were determined by an AMR-100 microplate reader (Allsheng Co, Ltd., Hangzhou, China) at OD_570_ nm. The test was performed in triplicates. The percentage inhibition of the biofilm was calculated according to the following equation:

$$\begin{array}{l} \text{Inhibition of biofilm }\left(\text{\%}\right)\\ \;=100-\frac{\left(\text{OD}570\text{ of wells in the treatment group x }100\right)\;}{\left(\text{OD}570\text{ of wells in the control group}\right)} \end{array}$$

### Effects of CFS of Lactic Acid Bacteria on Dispersal of Biofilm

The effect of CFS of lactic acid bacteria was determined on the dispersion of the preformed biofilm of *E. coli*. The biofilm was developed in a microtiter plate by adding 200 μl of 10^6^ CFU/ml of *E. coli* suspension and incubated at 37°C for 24 h. Following incubation, non-adherent cells were removed gently without disrupting the biofilm construction and washed with sterile DW before adding 200 μl of non-neutralizing CFS of lactic acid bacteria. The microtiter plate was incubated at 37°C for 2 h before performing a crystal violet staining assay as described above. The experiments were carried out in triplicates.

### Scanning Electron Microscopy for Biofilm Production

All biofilm specimens of the planktonic stage and the sessile stage were examined with a scanning electron microscope as described elsewhere, with minor modifications (27). For the planktonic stage, *E. coli* P01 was mixed with non-neutralizing or neutralizing CFS of P72N, while for the mature stage, it was mixed with non-neutralizing CFS of L25F in a sterile 24-well microtiter plate (Corning, Corning, NY, USA) with 12 mm round cover glass (no. 1 thickness; Electron Microscopy Sciences, Hatfield, PA, USA) and left incubated for 24 h at 37°C. *E. coli* P01 in a sterile MRS broth was used as a control for both stages. After incubation, the microtiter plate was gently washed to remove the non-adherent cells before fixation with glutaraldehyde. Dehydration of cover glass was performed by ethanol before drying with a critical point dryer (Leica EM CPD300, Leica Microsystems, Wetzlar, Germany). The cover glass was coated with gold in a Balzers SCD 040 sputter coater (Balzers Union Ltd., Balzers, Germany) before photographing with a scanning electron microscope (JSM-IT500HR, JEOL, Akishima, Japan).

### Statistical Analysis

The Mann–Whitney *U*-test was performed to compare the transfer frequencies of each treatment, and an independent *t*-test was conducted to analyze the relation of biofilm formation between control and CFS of lactic acid bacteria by using SPSS version 22 for Windows (IBM, Armonk, NY, USA). The significant difference was defined at *P* < 0.05.

## Results

### Preparation of CFS Dilution

**Figure 1:**
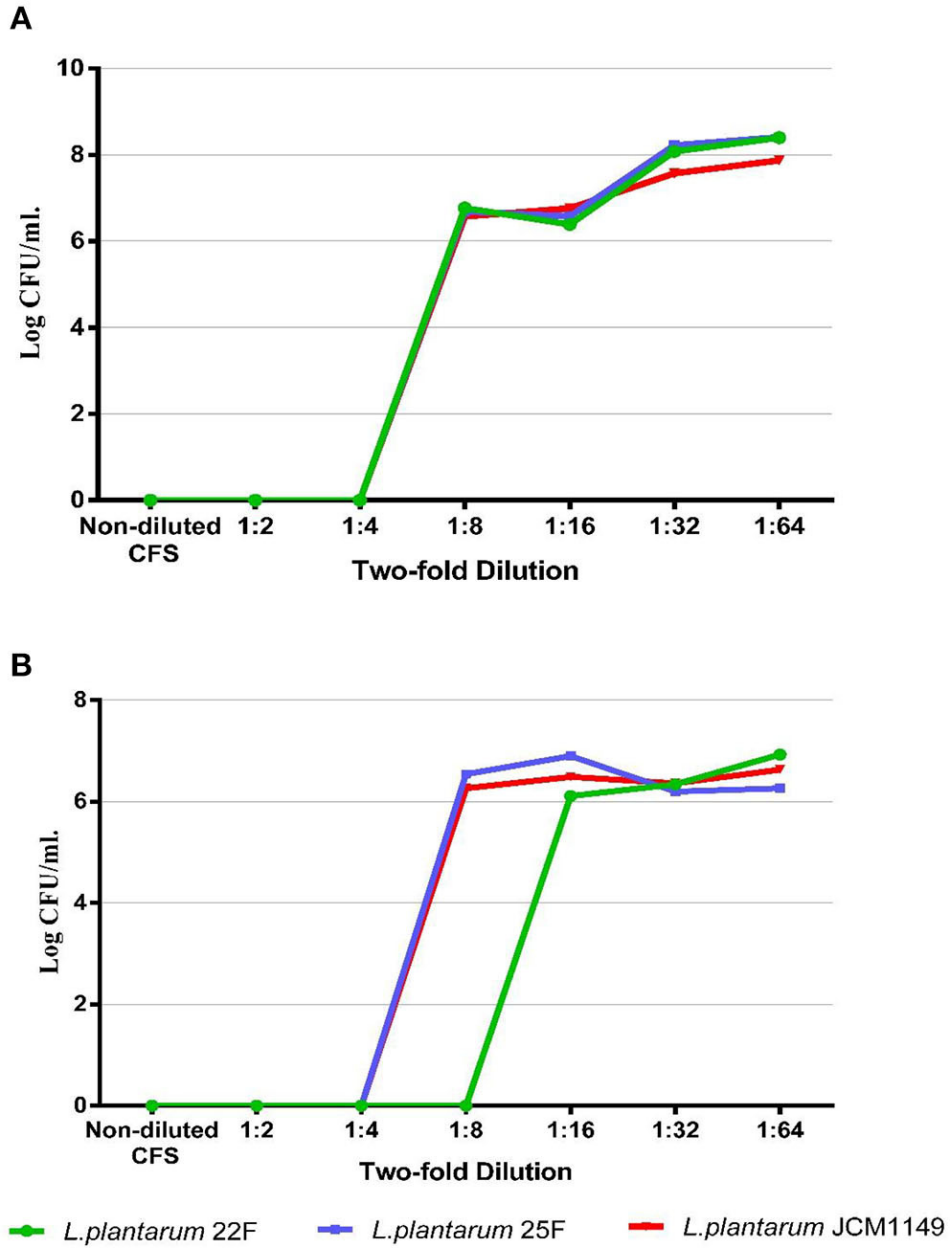
Bacterial survival of representative donor strain **(A)** and recipient strain **(B)** after culture with non-diluted and diluted CFS (non-diluted to 1:64) produced from selected LAB.

To determine the proper non-toxic CFS concentration that inhibits the conjugation, the bactericidal activity of serial diluents of LAB-CFS was evaluated against donor and recipient *E. coli* strains using the microdilution method. The proper dilutions that allowed the growth of donor and recipient *E. coli* are shown in Figures 1A,B, representatively. The CFS at dilutions of ≥1:16 showed no bactericidal activity against the tested strains, while the strong inhibition was still observed with lower dilutions at 1:4 and 1:8, proposing that the 1:16 dilution was a good candidate for further experiments.

### Anticonjugation Effect of CFS

The CFSs were evaluated for their anticonjugation effect on six colistin-resistant *E. coli* strains. A significant decrease in the transfer frequencies of colistin resistance gene *mcr*-1 was observed in the presence of non-neutralizing CFS (Table 2). The CFS (1:16) of *L. plantarum* 22F, 25F, and *P. acidilactici* 72N decreased the gene transfer frequency up to 100 times compared to the control. Interestingly, *L. plantarum* 22F significantly reduced the transfer frequencies in all colistin-resistant *E. coli* isolates (*P* <0.05). The transconjugants or recipient *E. coli* J53 receiving colistin-resistant gene from donor *E. coli* strains were confirmed by the presence of *mcr*-1 gene using PCR, broth microdilution assay, and plasmid replicon typing. The results showed that transconjugants acquired the *mcr*-1 gene, colistin resistance, and three plasmid replicon types of FIB, Frep, and Y (Table 3).

**Table 2:** Effects of CFS of LAB on transfer frequency.

***E. coli* strains**	**Treatments**							
	**Control**	**L22F (di1ution at 1:16)**	**L22F (neutralize condition)**	**L25F (di1ution at 1:16)**	**L25F (neutralize condition)**	**P72N (di1ution at 1:16)**	**P72N (neutralize condition)**	***E. coli* ATCC 25922**
P01	8.67 × 10^−4*b*^	3.90 × 10^−5*a*^	9.56 × 10^−4*b*^	4.38 × 10^−5*a*^	1.04 × 10^−3*b*^	4.95 × 10^−5*a*^	1.05 × 10^−3*b*^	1.10 × 10^−3*b*^
P02	8.63 × 10^−5*d*^	2.86 × 10^−5*a*^	2.41 × 10^−4*b*^	4.95 × 10^−5*ad*^	2.53 × 10^−4*bc*^	3.24 × 10^−5*a*^	4.48 × 10^−4*b*^	2.92 × 10^−4*b*^
H01	4.28 × 10^−4*c*^	4.86 × 10^−5*a*^	6.92 × 10^−4*b*^	1.37 × 10^−4*a*^	6.01 × 10^−4*bc*^	3.24 × 10^−4*abc*^	7.27 × 10^−4*b*^	7.38 × 10^−4*b*^
H02	3.37 × 10^−4*cd*^	1.62 × 10^−5*a*^	3.14 × 10^−4*cd*^	1.29 × 10^−4*ace*^	8.36 × 10^−4*bd*^	2.95 × 10^−5*e*^	3.96 × 10^−4*d*^	7.21 × 10^−4*d*^
E01	4.48 × 10^−4*d*^	1.42 × 10^−4*a*^	1.04 × 10^−3*c*^	1.86 × 10^−4*a*^	9.75 × 10^−4*bc*^	1.29 × 10^−4*a*^	9.93 × 10^−4*c*^	1.01 × 10^−3*c*^
E02	1.05 × 10^−3*b*^	1.71 × 10^−4*a*^	1.30 × 10^−3*b*^	2.11 × 10^−4*a*^	1.28 × 10^−3*b*^	3.05 × 10^−4*a*^	1.18 × 10^−3*b*^	1.42 × 10^−3*b*^

*The different lowercase letters within the row indicate significant differences between treatments (P < 0.05) determined by Mann–Whitney U-test*.

**Table 3:** The characteristics of transconjugants after treatment with CFS of LAB.

**Donor *E. coli* strains**	**Recipient** ***E. coli*** **J53 (transconjugants)**		
	**colistin MIC**	***mcr*-1**	**Plasmid**
	**(μg/ml)**	**gene**	**replicon**
P01	8	+	Frep
P02	16	+	Frep
H01	4	+	FIB
H02	8	+	FIB, Frep
E01	4	+	FIB, Y
E02	4	+	FIB

### Assessment of the Antibiofilm Activity of LAB-CFS Against Planktonic and Sessile Stages of *E. coli*

**Figure 2:**
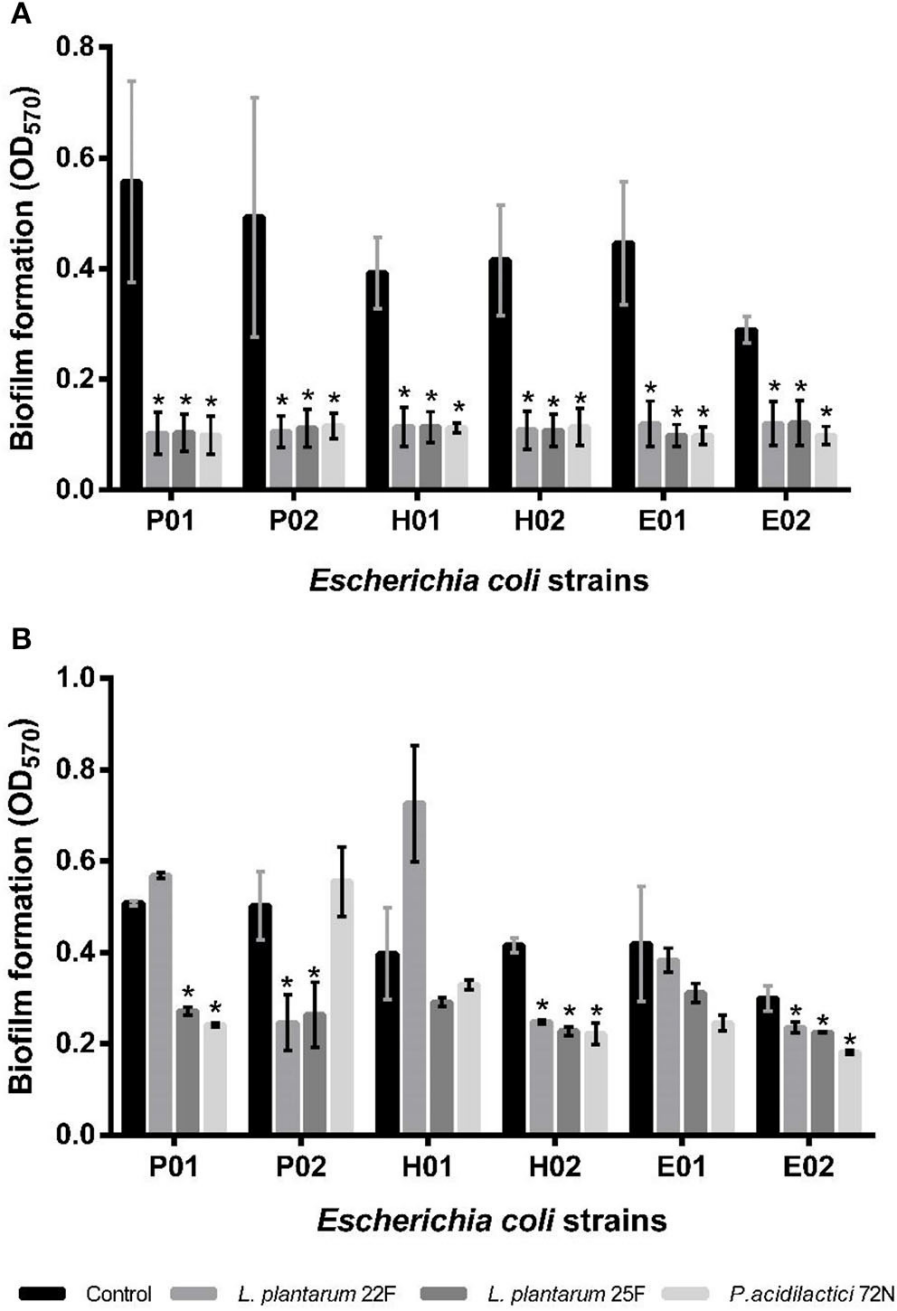
Effects of non-neutralizing CFS **(A)** and neutralizing CFS **(B)** of LAB on biofilm of *E*. *coli* evaluated by crystal violet assay. A significant difference (*P < 0.05) was calculated by an independent *t*-test when compared with the control group.

During the planktonic stage, all of our non-neutralizing CFSs significantly decreased (*P* < 0.05) the biofilm formation of all tested *E. coli* strains (Figure 2A). *P. acidilactici* 72N demonstrated the highest reduction in biofilm formation; however, the percentage of inhibition induced by other LAB-CFS ranged between 50.20 and 82.28% (Supplementary Table 1). For the neutralizing CFS (pH 6.5), the CFS of *L. plantarum* 25F exhibited the highest potential toward the antibiofilm activity of the tested *E. coli* isolates (Figure 2B). Nevertheless, the maximum percentage inhibition (52.59%) was observed after the treatment with *P. acidilactici* 72N CFS, while other LAB-CFS showed variable degrees of inhibition that ranged between 0 and 51.03% (Supplementary Table 2).

**Figure 3:**
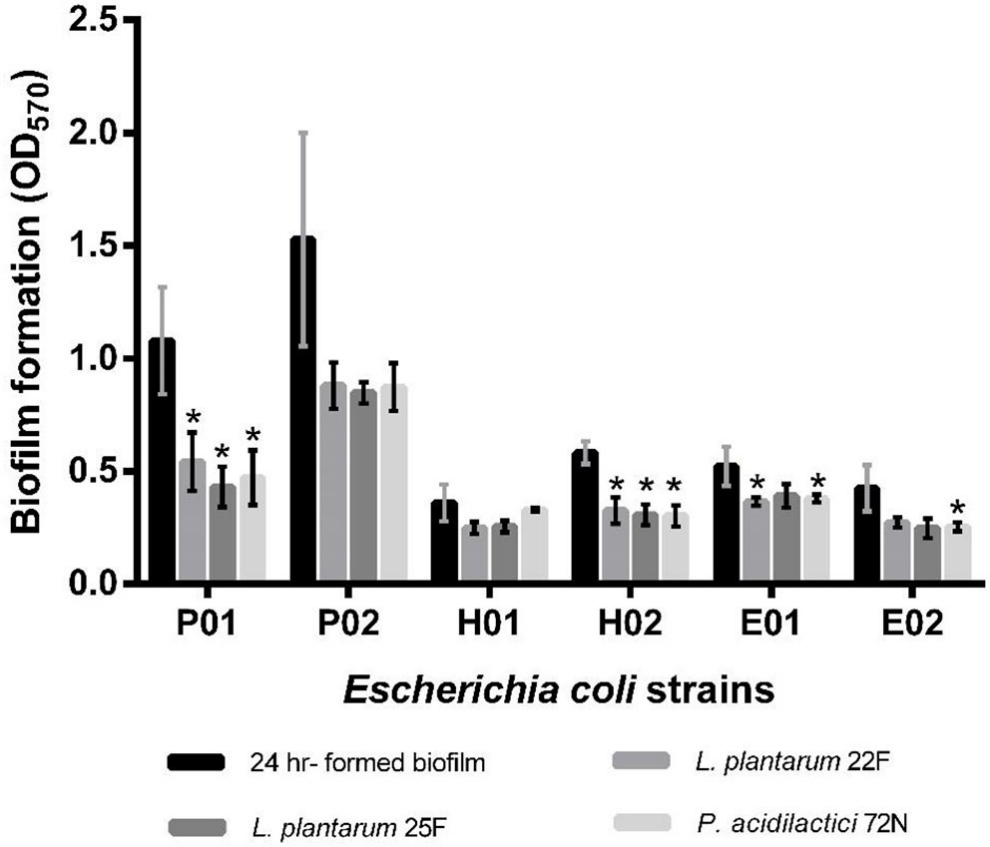
Effects of non-neutralizing CFS of LAB on sessile biofilm of by *E. coli* evaluated by crystal violet assay. A significant difference (*P < 0.05) was calculated by an independent *t*-test when compared with the control group.

Regarding the sessile stage, the antibiofilm activity of different neutralizing CFSs of LAB against *E. coli* strains is presented in Figure 3. As indicated, *P. acidilactici* 72N CFS significantly decreased the sessile biofilms formation against most of the tested *E. coli* strains. Similarly, *L. plantarum* 25F CFS induced a substantial reduction in *E. coli* adherence and biofilm production. The percentage inhibition of biofilm was 60.10%, in the case of *L. plantarum* 25F CFS, while it ranged from 8.38 to 56.34% in other LAB-CFS (Supplementary Table 3).

**Figure 4:**
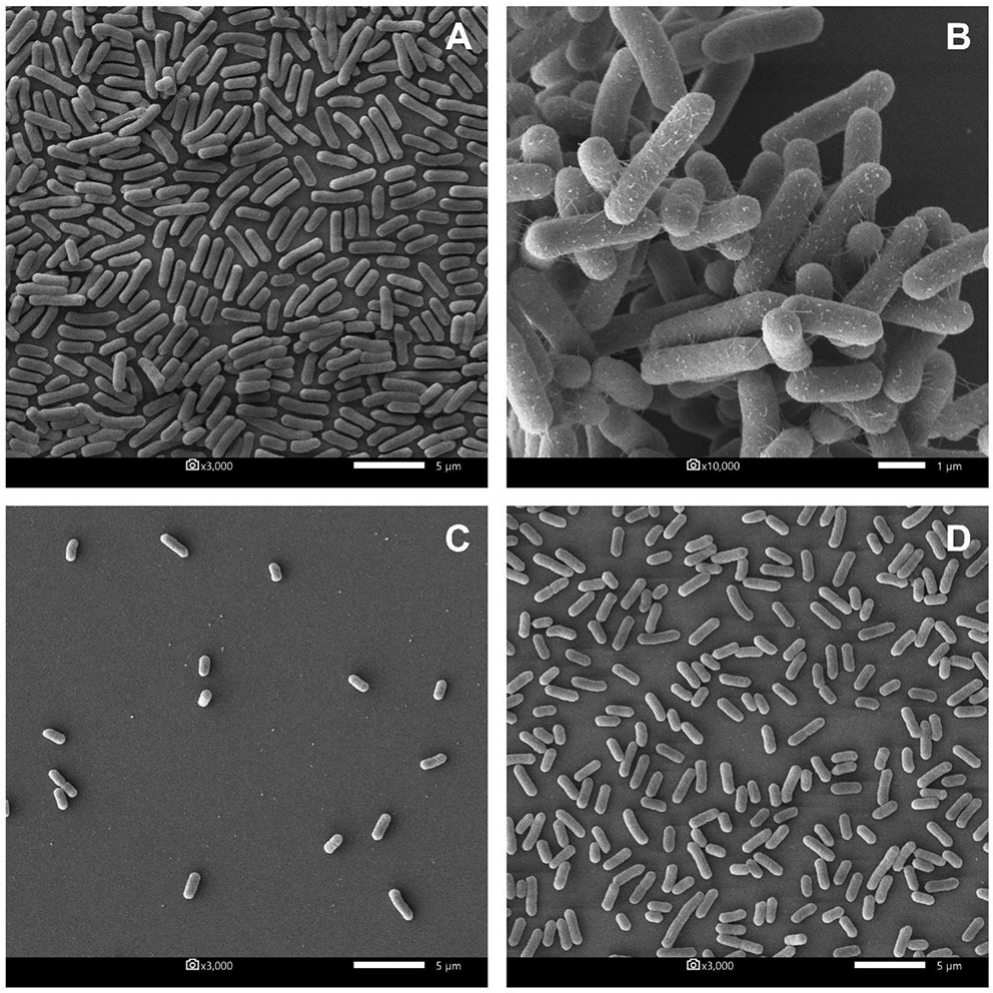
Scanning electron micrographs of biofilm formed by *E. coli* P01 in planktonic stage with different conditions. **(A,B)** Represent *E. coli* grew toward Sterile MRS broth (magnification: 3000X and 10,000X, respectively). **(C,D)** represent *E. coli* cultured in non-neutralizing and neutralizing CFS of P72N, respectively (magnification: 3000X). Scale bars are 1 or 5 μm.

**Figure 5:**
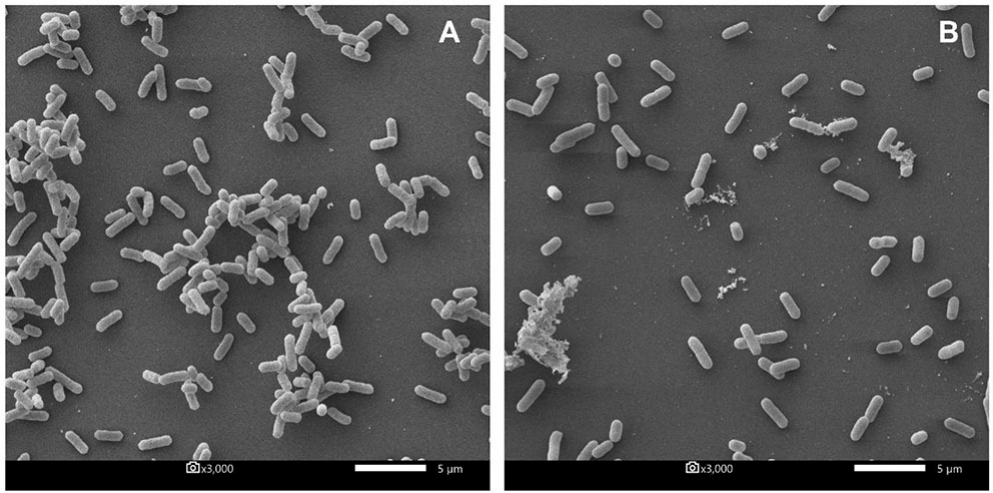
Scanning electron micrographs of biofilm formed by *E. coli* P01 in sessile stage with different conditions. **(A)** Represents *E. coli* cultured into sterile MRS broth for 24 h. **(B)** Represents 24 h-biofilm of *E. coli* after exposure to non-neutralized CFS of L25F for 2 h. Scale bar is 5 μm, and magnification is 3000X.

Furthermore, the biofilm illustration produced by *E. coli* P01 during the planktonic stage was confirmed by SEM. It was observed that the control sample (*E. coli* P01 cultured into sterile MRS broth) showed high cell density, aggregation (Figure 4A), and extracellular polysaccharide (EPS) matrix (Figure 4B). Compared to the control, the non-neutralizing CFS of P72N substantially reduced the adherence and aggregation of tested *E. coli* strain after 24 h of incubation (Figure 4C). Moreover, the neutralizing CFS of P72N also demonstrated the low cell density and the EPS matrix against the tested *E. coli* strain (Figure 4D). On the other hand, the scanning electron micrographs of the biofilm formed by *E. coli* P01 during the sessile stage are illustrated in Figure 5. It appeared that the low cell density, aggregation of the tested strain, and the EPS matrix were obviously reduced, in which they grew in non-neutralizing CFS of L25F for 2 h (Figure 5B), while the *E. coli* P01 cultured into sterile MRS broth for 24 h (Figure 5A). These results are in agreement with the findings of the crystal violet technique, which showed a significant reduction in bacterial adherence and biofilm formation (Figures 2, 3).

## Discussion

Probiotics, especially lactobacilli, have received significant attention because of the growing evidence of health benefits associated with their use. In our previous studies, *L. plantarum* 22F, 25F, and *Pediococcus acidilactici* 72N were characterized based on acid, bile, and temperature tolerance, good survivability, and absence of antibiotic-resistant genes (18). Furthermore, they displayed promising bactericidal capacity against several bacterial pathogens as well as antiviral activity against PEDV (19, 20). The emergence of plasmid-mediated colistin resistance and biofilm formation among different pathogens has increased global awareness and concerns. In the present study, we reported for the first time the beneficial role of LAB strains on antibiotic resistance gene transfers and biofilm formation in six *E. coli* strains. Our results clearly supported the antibiofilm and anticonjugation role of the LAB strains against *E. coli* harboring the *mcr*-1 gene. *E. coli* isolated from different origins (animal, farmer, and farm environment) with different characteristics such as variable degrees of colistin resistance, antibiotic susceptibility, plasmid replicon types, and biofilm formation. Thus, *E. coli* isolates in this study may be good and representative candidates for antimicrobial study.

Lactic acid bacteria generally secrete many inhibitory substances such as bacteriocins, fatty acids, and organic acids (lactic and acetic acids). These inhibitory compounds can directly disrupt the bacterial outer membrane leading to cell death (28–30). Therefore, the optimum non-bactericidal dilution of CFS was determined prior to anticonjugation and antibiofilm experiments. We found optimum dilution of CFS (1:16) that was non-inhibitory to bacterial growth, however, yet maintained the strong anticonjugation and antibiofilm activity. Colistin resistance encoded by the *mcr-*1 gene is mostly harbored on a conjugative plasmid, which facilitates its transfer to other bacteria through horizontal gene transfer (3). Conjugation generally transfers mobile genetic elements such as a plasmid, integrative and conjugative element, or pathogenicity islands between donor and recipient cells through direct physical contact *via* sex pilus or nanotubes (31).

In this study, all non-neutralizing LAB-CFS significantly decreased the transfer frequencies of colistin resistance gene *mcr*-1; however, neutralizing CFS failed to show any anticonjugation activity. These findings were consistent with the results described in the previous report of *Bifidobacteria* in decreasing β-lactam resistance gene transfer (*bla* genes) among *Enterobacteriaceae* (14). Inhibition of conjugation has been described with the agents that affect the formation of sex pili or allow plasmid curing of donor strains (32). El-Deeb et al. (15) reported plasmid curing activity of *B. longum, L. plantarum*, and *S. thermophilus* against multidrug-resistant bacterial isolates (MDRs). It was assumed that certain chemicals present in CFS may interfere with plasmid DNA replication *via* blocking the DNA gyrase activity (33). Unsaturated fatty acids, including linoleic, oleic, and stearic acid secreted by LAB, have also been proposed as one of the inhibitory compounds of conjugation. These fatty acids inhibited the activity of the plasmid-encoded type IV traffic ATPase (TraW). TraW regulates the switching between DNA translocation and pilus biogenesis through the conjugation machinery (34, 35). Even though an increasing number of *mcr*-like genes [*mcr-*2 (36), *mcr*-3 (37, 38), *mcr-*4 (39), *mcr*-5 (40), *mcr-*6 (41, 42), *mcr*-7 (43), *mcr-*8 (44), *mcr*-9 (45), and *mcr*-10 (5)] have been identified yet, however, given the common mechanism of conjugation, our LAB strains may decrease the transfer frequencies of other plasmid-encoded colistin resistance genes.

Bacterial biofilms are known as sessile microbial communities that are attached to the surface and mostly embedded in a self-produced matrix of organic polymers. Bacteria in biofilms are more resistant to antibiotics, disinfectants, dying, and dynamic environments. Antibiotics are of limited use against biofilms as most of the antibiotics are only active against planktonic microorganisms and cannot disperse biofilms. Targeting biofilm formation is a promising target for therapeutic intervention, which has gained significant attention in the last few decades and encouraged the discovery of biofilm inhibitors (46, 47). Indeed, several antibiofilm compounds do not have any antimicrobial properties against planktonic cells. Hence, it is necessary to evaluate the potential effects of our LAB strains on biofilm formation in both planktonic and sessile stages (7). The results showed that the tested LAB strains were able to reduce the biofilm formation in both planktonic and sessile states of *E. coli*. Biofilm formation in contact with non-neutralizing CFS reduced about 82.2% compared to the control. Interestingly, the neutralizing CFS also reduced the biofilm formation up to 52%, despite having no bactericidal activity (20). Similar findings of biofilm reduction have been reported in other bacterial pathogens, where 50–57% reduction in the biofilm formation of *Vibrio cholerae, E. coli*, and *S. aureus* was observed by the neutralizing CFS of lactobacilli isolates (26, 48). In contrary, Chapman et al. (49) reported no antibiofilm activity of neutralizing lactobacilli-CFS against *E. coli* NCTC 9001 and *E. faecalis* NCTC 00775. Thus, it could be attributed to the different origins and characteristics of LAB isolates and tested pathogens.

Indeed, there is no specific mechanism by which LAB prevents the biofilm formation; however, several studies have proposed that probiotics can influence the expression of genes involved in quorum sensing, cell adhesion, virulence factors, and the formation of biofilms (50). LAB also secretes a variety of extracellular inhibitory substance, which includes extracellular substance (16), exopolysaccharides (17), biosurfactants (51, 52), bacteriocins (53), different enzymes (54), and antiquorum compounds (55, 56). Specifically, several studies have reported that bacteriocin may decrease the formation of biofilms due to growth inhibition. However, the neutralizing CFS of our LAB strains showed no antibacterial activity, proposing that bacteriocin may not have caused biofilm inhibition in this study (20).

The average pH in the pig's intestine ranges from 6.0 to 6.7, but LAB could acidify the intestine conditions by producing different organic acids (57, 58). In this study, the non-neutralizing CFS (pH in the range 3.70–3.98) markedly reduced biofilm formation up to 82%, whereas the antibiofilm activity of neutralizing CFS (pH: 6.5) was also decreased (up to 60%); moreover, the significant inhibition was still observed when compared to the control. Similar findings have been reported earlier, where the lactobacilli-CFS dispersed the sessile biofilm of *Vibrio cholerae* between 62 and 85% in the non-neutralizing form and between 50 and 75% in the neutralizing form (26). However, their results showed a non-significant difference between the neutralizing and pH neutralizing CFS to biofilm dispersal effect suggesting that the inhibition of biofilm formation by lactobacilli CFS was not due to its antimicrobial activity but to the CFS component, such as certain disintegrative enzymes, which need to be proven in further studies. Simultaneously, SEM analysis in our study showed low aggregation of *E. coli* cells in the biofilm after treatment with LAB-CFS. This suggests the active role of certain metabolites such as enzymes or dispersal signal molecules that may have contributed to biofilm inhibition (59, 60). Overall, the current study gave insight into the potential role of LAB-CFS on the biofilm reduction and growth inhibition of *E. coli*. However, further study is still urgently needed to fully understand the molecular mechanisms responsible for the anticonjugation and antibiofilm activity of LAB.

In conclusion, the present study showed the ability of LAB isolates to produce antimicrobial compounds that inhibit bacterial conjugation and limit the dissemination of antibiotic resistance genes. The biofilm formed by colistin-resistant *E. coli* was successfully removed by the cell-free supernatants of LAB, proving that LAB can serve as a potential alternative to antibiotics.

## Data Availability Statement

The original contributions presented in the study are included in the article/Supplementary Materials, further inquiries can be directed to the corresponding author.

## Author Contributions

PA conceived and designed the experiments, performed the experiments, analyzed the data, and contributed to the writing of the manuscript. PP performed the experiments and analyzed the data. JY performed the experiments. WN, WS, and KL contributed reagents, materials, and analysis tools. AS contributed to the writing of the manuscript. NP conceived and designed the experiments, analyzed the data, and contributed to the writing of the manuscript. All authors contributed to the article and approved the submitted version.

## Conflict of Interest

The authors declare that the research was conducted in the absence of any commercial or financial relationships that could be construed as a potential conflict of interest.
