# Supplementary material for: Anticonjugation and Antibiofilm Evaluation of Probiotic Strains Lactobacillus plantarum 22F, 25F, and Pediococcus acidilactici 72N Against Escherichia coli Harboring mcr-1 Gene
Source: Front Vet Sci. 2021 Jun 11;8:614439. doi: 10.3389/fvets.2021.614439 (PMC8225926; doi:10.3389/fvets.2021.614439)
Supplement: Supplementary file 1 [file Data_Sheet_1.docx]

SUPPLEMENTARY MATERIAL

**Anticonjugation and Antibiofilm Evaluation of Probiotic Strains *Lactobacillus plantarum* 22F, 25F, and *Pediococcus acidilactici* 72N Against *Escherichia coli* Harboring *mcr*-1 Gene**

**Prasert Apiwatsiri^1^, Pawiya Pupa^1^, Jitrapa Yindee^1^, Waree Niyomtham^1^, Wandee Sirichokchatchawan^2^, Kittitat Lugsomya^3^, Asad Ali Shah^1^ and Nuvee Prapasarakul^1,4*^**

^1^ Department of Veterinary Microbiology, Faculty of Veterinary Science, Chulalongkorn University, Bangkok, Thailand,

^2^ College of Public Health Sciences, Chulalongkorn University, Bangkok, Thailand,

^3^ Jockey Club College of Veterinary Medicine and Life Sciences, City University of Hong Kong, Kowloon Tong, Hong Kong,

^4^ Diagnosis and Monitoring of Animal Pathogens Research Unit, Chulalongkorn University, Bangkok, Thailand

Nuvee Prapasarakul
Nuvee.p@chula.ac.th

**TABLE S1│** Percentage inhibition of planktonic biofilm formation of *E. coli* by non-neutralized CFS producing by lactic acid bacteria. The results express as means ± the standard errors. The experiment was performed in triplicates.

| **Lactic acid bacteria** | ***Escherichia coli*** | | | | | |
| --- | --- | --- | --- | --- | --- | --- |
|  | **P01** | **P02** | **H01** | **H02** | **E01** | **E02** |
| *L. plantarum* 22F | 81.83±1.78 | 77.29±6.71 | 77.22±6.71 | 74.36±6.71 | 73.58±3.42 | 50.54±2.95 |
| *L. plantarum* 25F | 81.29±3.93 | 76.31±6.11 | 71.18±4.40 | 74.22±1.82 | 77.69±2.19 | 50.20±3.99 |
| *P. acidilactici* 72N | 82.28±1.41 | 74.95±1.41 | 71.02±3.78 | 72.65±3.93 | 77.48±2.51 | 57.68±6.91 |

**TABLE S2│** Percentage inhibition of planktonic biofilm formation of *E. coli* by neutralized CFS producing by lactic acid bacteria. The results express as means ± the standard errors. The experiment was performed in triplicates. Zero indicates that CFS of tested LAB showed no biofilm inhibition.

| **Lactic acid bacteria** | ***Escherichia coli*** | | | | | |
| --- | --- | --- | --- | --- | --- | --- |
|  | **P01** | **P02** | **H01** | **H02** | **E01** | **E02** |
| *L. plantarum* 22F | 0 | 51.03±12.20 | 0 | 40.24±1.10 | 8.37±6.13 | 21.18±3.58 |
| *L. plantarum* 25F | 46.49±1.72 | 47.51±14.10 | 26.62±2.39 | 45.14±2.38 | 25.60±5.02 | 24.86±0.39 |
| *P. acidilactici* 72N | 52.59±0.97 | 0 | 17.21±2.72 | 46.67±5.58 | 41.31±3.87 | 39.46±1.53 |

**TABLE S3│** Percentage inhibition of sessile biofilm formation of E. coli by non-neutralized CFS producing by lactic acid bacteria. The results express as means ± the standard errors. The experiment was performed in triplicates.

| **Lactic acid bacteria** | ***Escherichia coli*** | | | | | |
| --- | --- | --- | --- | --- | --- | --- |
|  | **P01** | **P02** | **H01** | **H02** | **E01** | **E02** |
| *L. plantarum* 22F | 49.76±12.02 | 42.41±6.79 | 31.04±7.25 | 43.99±10.79 | 29.92±3.68 | 35.63±5.36 |
| *L. plantarum* 25F | 60.10±8.29 | 44.51±3.10 | 29.21±7.12 | 47.30±7.93 | 25.15±9.94 | 41.62±10.19 |
| *P. acidilactici* 72N | 56.34±11.18 | 42.82±6.95 | 8.38±2.20 | 48.04±8.04 | 27.41±3.44 | 40.34±4.59 |
